# Evaluation of the Optic Nerve Head in Glaucoma

**DOI:** 10.5005/jp-journals-10008-1146

**Published:** 2013-09-06

**Authors:** Monica Gandhi, Suneeta Dubey

**Affiliations:** Consultant, Department of Glaucoma and Anterior Segment, Dr Shroff’s Charity Eye Hospital, New Delhi, India; Consultant, Department of Glaucoma and Anterior Segment, Dr Shroff’s Charity Eye Hospital, New Delhi, India

**Keywords:** Optic disk, Optic cup, Neuroretinal rim, Optic disk hemorrhage, Retinal nerve fiber layer, Parapaillary atrophy, Disk anomalies.

## Abstract

Glaucoma is an optic neuropathy leading to changes in the intrapaillary and parapaillary regions of the optic disk. Despite technological advances, clinical identification of optic nerve head characteristics remains the first step in diagnosis.

Careful examination of the disk parameters including size, shape, neuroretinal rim shape and pallor; size of the optic cup in relation to the area of the disk; configuration and depth of the optic cup; ratios of cup-to-disk diameter and cup-to-disk area; presence and location of splinter-shaped hemorrhages; occurrence, size, configuration, and location of parapapillary chorioretinal atrophy; and visibility of the retinal nerve fiber layer (RNFL) is important to differentiate between the glaucomatous and nonglaucomatous optic neuropathy.

**How to cite this article:** Gandhi M, Dubey S. Evaluation of the Optic Nerve Head in Glaucoma. J Current Glau Prac 2013;7(3):106-114.

## INTRODUCTION

Glaucoma is a chronic progressive optic neuropathy described by the morphological changes in the intrapapillary and parapapillary regions of the optic nerve head and the retinal nerve fiber layer (RNFL). Appropriate techniques of clinical evaluation can help differentiate between glaucomatous, nonglaucomatous and normal optic disks. The various aspects to observe are the optic disk size and shape, cup-to-disk (C:D) ratio in relation to the disk size, configuration and depth of the optic cup, the configuration of the neuroretinal rim, position of the exit of the central retinal vessel trunk, presence and location of disk hemorrhage, RNFL defects, and configuration and location of parapapillary chorioretinal atrophy.

The observations can be documented in form of photographs and discograms. The prognostic importance of each parameter is important to understand. The identification of physiological variations, disk anomalies and appropriate classifications of the optic nerve are integral to complete the evaluation.

### Optic Disk Size

The size of the normal optic disk shows interindividual variability. In Indian eyes the mean optic disk area measured 2.25 ± 0.51 mm^2^ as reported in Central India Eye and Medical study^1^ (CIEMS). The South Indian population [Vellore Eye Study (VES)]^2^ showed mean area of 2.58 mm^2^. In the Andhra Pradesh study,^3^ however, the mean optic disk area was 3.37 mm^2^. The differences may be due to the techniques used to determine size. In a Caucasian population the reported range is 0.80 to 5.54 mm^2^.^4^

The disk area has been significantly correlated with axial length and refractive error in certain studies^1,5,6^ whereas no correlation was reported in the VES.^2^ The size is independent of age beyond 10 years. No conclusive correlation has been seen with gender, body height, best corrected visual acuity (BCVA) and anterior chamber depth.

The exact relationship between the optic disk size and glaucoma is not clear. Jonas et al^7^ reported higher susceptibility of neuroretinal rim loss in area farthest from the exit of central retinal vessel trunk, which is greater in a large disk. Afro-Caribbean subjects are known to have larger disks and greater susceptibility to glaucoma as compared to Caucasian population.

Other optic nerve pathologies may have an association with disk sizes as optic disk drusen, pseudopapilledema, and nonarteritic ischemic optic neuropathy are seen in smaller optic disks. Normal tension glaucoma, pits of the optic nerve head and morning glory syndrome are more commonly associated with large optic nerve heads.

Clinically it is important to determine if the optic disk is of an average size, larger or smaller than average for the given population. A normal disk can be of a small size and a normal disk can be of a larger size; size by itself does not determine glaucoma. It is relevant because the disk size determines the C:D ratio and the neuroretinal rim thereof.^4^ In a large disk one would expect a large cup and a large neuroretinal rim and in a small disk there is usually no cup.

### Determination of Size

**Fig. 1:**
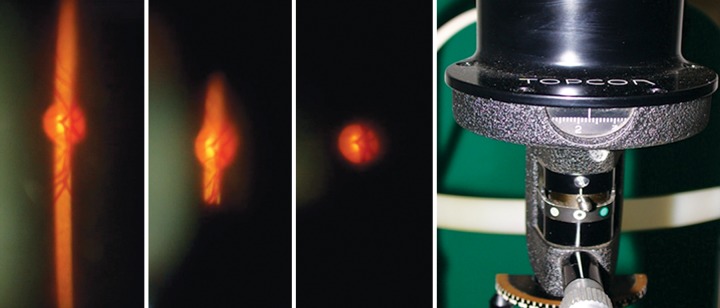
To determine disk size decrease the height of the slit beam to coincide with the disk margin.

On the slit lamp, examine the optic disk by looking at it stereoscopically through noncontact lenses like +60 D, +78 D, +90 D or Super field NC. There is better interobserver agreement in size determined through a dilated pupil.^8^ To determine the size, reduce the height of the slit beam to coincide with the disk margins and then read the measurements from the graticule on the slit lamp. And then measure the horizontal diameter (Fig. 1). This measurement would need to be corrected as the size of the image would depend on the magnification properties of the eye and the instruments.^9,10^

Volk

60 D              0.88

78 D              1.2

90 D              1.33

Super field NC 1.5

Nikon

60 D              1.03

90 D              1.63

Read the measurement from the scale

**Fig. 2A:**
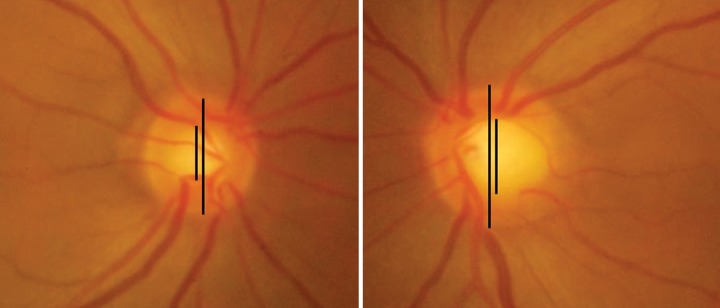
Asymmetry in disk size leading to asymmetry in CD ratio. Left disk is larger

**Fig. 2B:**
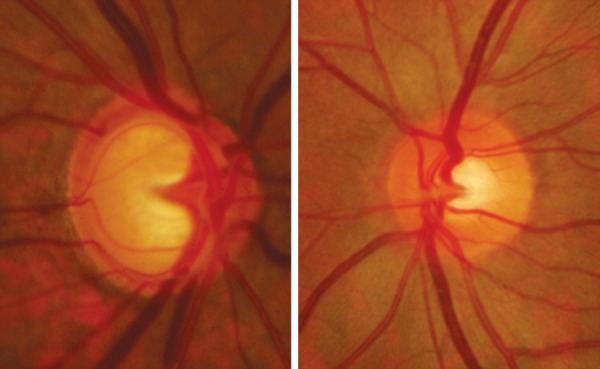
Note the difference in 2 eyes of same patient. The right eye is larger with larger CD ratio and thinner NRR. Asymmetry in CD ratio is due to disk size and also glaucomatous Involvement of the NRR

To define the borders of the optic disk look for the thin white band encircling the disk. This is the inner side of the peripapillary scleral ring and is more easily detected on the temporal side and when viewing the disk stereoscopically.

With ophthalmoscope – using the technique described by Gross^11^ the 5° aperture of the Welch Allyn ophthalmoscope is used to project a circular spot close to the optic disk and the size of the disk is compared with the circle. The circle has a diameter of 1.5 mm and an area of 1.77 mm^2^ which is slightly smaller than the average disk. With this one can determine if the disk is small, average or large in size.

Compare the disk sizes of both the eyes and look for asymmetry. The asymmetry may be due to actual difference in size or due to the refractive status of the eye or the axial length. It is possible that the difference in disk sizes account for the asymmetry in other parameters like C:D ratio and the neuroretinal rim (Figs 2A and B).

### Optic Disk Shape

**Fig. 3:**
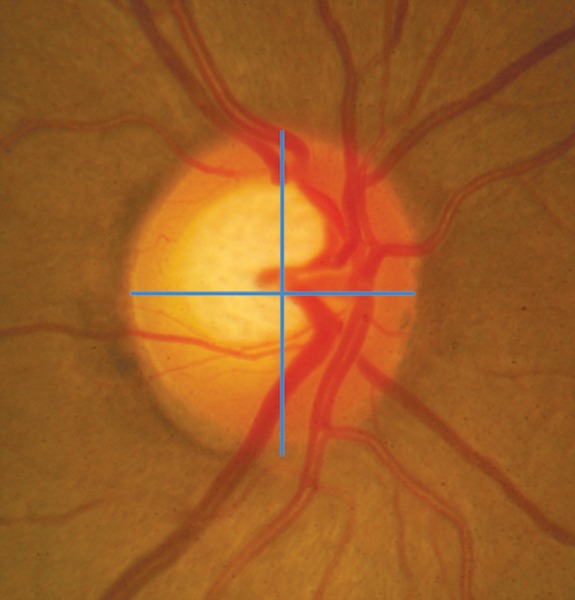
Vertically oval disk. Vertical diameter longer than horizontal diameter

**Figs 4A to c:**
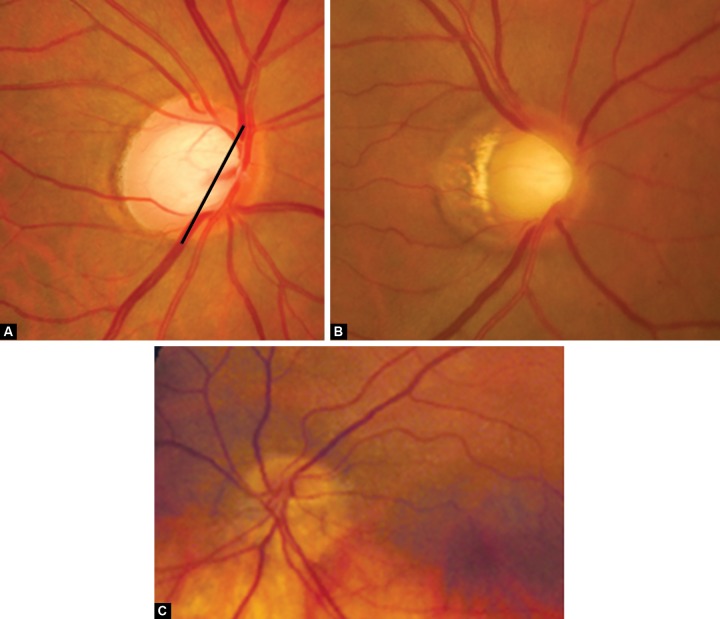
(A) Torted disk, (B) tilted disk, (C) tilted bean shaped disk

A normal optic disk is vertically oval with the vertical diameter being the maximum diameter and the horizontal being the minimum diameter (Fig. 3).The vertical axis is about 6% longer than the horizontal in Indian eyes^2^ and 9% in a Caucasian population.^4^ In 81.4% Indian eyes (VES), the vertical diameter was longer than the horizontal axis, in 14.3% eyes the horizontal optic disk was longer than the vertical and in 4.2% the vertical and horizontal diameters were equal. Apart from these it is not uncommon to see disks which are not vertically or horizontally oval (Figs 4A to C). The variations in shape may be accompanied with astigmatism and amblyopia.^12^ An optic disk is considered torted when the vertical axis of the optic disk is rotated >15° from the vertical meridian. A tilted optic disk is when there is (three-dimensional) angulation of the (anteroposterior) optic cup axis.

**Fig. 5:**
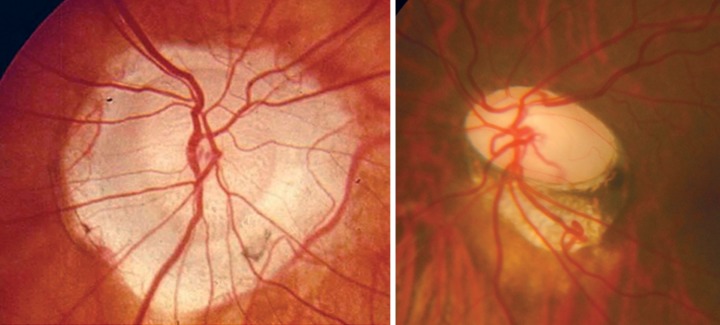
Variation in disk shape in myopes

It is important to determine unusual shapes and document them as it would help in future comparisons and also the ISNT rule (discussed later) will not always be applicable in disks which are not vertically oval. The clinical significance of the shape also lies in the fact that it infuences the distance between NRR at the disk border and the central retinal vessel trunk exit. Myopic eyes may have a variation in disk shape, and due to presence of peripapillary atrophy, determination of disk margin may be difficult (Fig. 5). With highly myopic eyes excluded, the optic disk shape as single variable is not markedly important for pathogenesis, early diagnosis and differential diagnosis of the glaucomas.

**Fig. 6:**
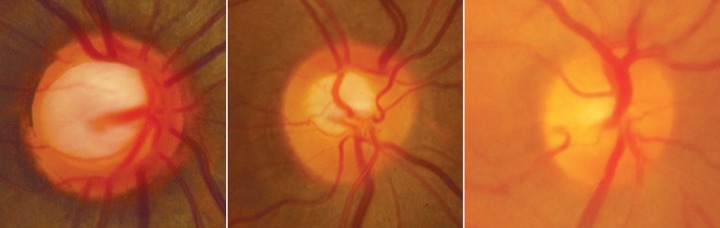
Large disk with large cup, average size disk with average sized cup and no cup in a small disk

### Cup Disk Ratio in Relation to the Disk Size

The size of the cup is determined by the size of the optic disk. There is a positive correlation between the vertical diameter and the vertical C:D ratio. A large disk will have a large cup, an average disk will have an averaged sized cup and a small disk will usually have no cup (Fig. 6). This fact is important to remember as early or even moderately advance glaucoma may be missed if we judge only using the C:D ratio.

Mean area of the optic cup in Indian eyes is 0.98 mm^2^. The area of the optic cup is independent of age, refractive error, and sex, axial length of the globe and depth of the anterior chamber.^2^ The cup is horizontally oval. The mean horizontal C:D diameter ratio is 0.66 and mean vertical C:D diameter ratio is 0.56.^2^

**Fig. 7:**
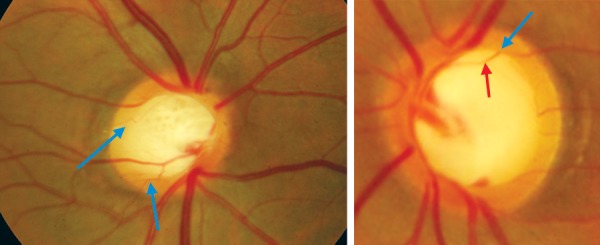
To determine the cup margin–the second kink (blue arrow) is the cup edge (the first kink in the vessel-red arrow)

The configuration and depth of the cup are best judged by the stereoscopic examination. Contour cup is more important than the color cup. Carefully follow the optic nerve blood vessels and note the kinks in the vessels as they climb on the cup wall. At the cup rim junction the vessel will kink and bend over the rim. This second kink in the blood vessel determines the cup margin (Fig. 7).

### The Neuroretinal Rim

The neuroretinal rim is the most important parameter of the optic disk evaluation. The optic disk is vertically oval and the cup is horizontally oval thus the rim has a characteristic configuration where the inferior (I) rim is the widest, followed by the superior (S) and nasal rims (N) and the temporal (T) rim is the thinnest. This is the ‘ISNT rule' which helps to determine glaucomatous changes in the disk glaucoma. On an average, the inferior rim is 18% thicker than the superior rim.^15^

The cardinal feature of glaucomatous optic neuropathy is the loss of NRR from the inner edge of the rim. This loss can occur in all sections of the disk with regional preference depending on the stage of glaucoma.^5^ The sequence of loss is usually first in the inferotemporal and superotemporal disk regions. So for early diagnosis these areas should be carefully evaluated for glaucomatous changes. In moderate disease the temporal part of the horizontal disk is involved and in advanced glaucoma the rim remnants are located mainly in the nasal sectors. This accounts for the changes seen in the visual fields in various stage of the disease with early perimetric changes in the upper nasal quadrant of the visual field and the area preserved till advanced disease is the inferotemporal Island of vision.^14^

**Fig. 8:**
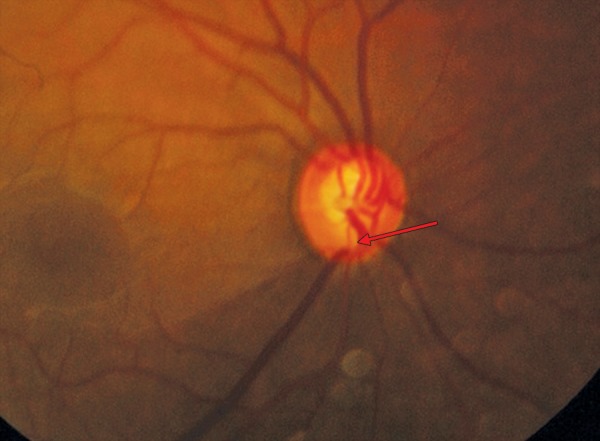
Inferior notch. Note the associated retinal nerve fiber layer defect

In Indian eyes the mean neuroretinal rim area is 2.29 ± 0.39 mm^2^ as reported by the Chennai Glaucoma study group.^15^ This is higher than that reported by Jonas et al^4^ among Caucasia subjects (1.97 ± 0.5 mm^2^). Reported rim areas among South Indians in VES^2^ was 1.6 ± 0.37 mm^2^, and 2.8 ± 0.53 mm^2^ in a study of 153 participants of the Andhra Pradesh Eye Diseases Study (APEDS).^3^

The rim area shows a strong positive correlation with the disk area. For every 1 mm^2^ increase in disk area, the rim area increased by 0.5 mm^2^.^15^ The larger is the optic nerve the larger is the NRR area and as the glaucoma progresses there is an increase in the cup area thus correspondingly the NRR area decreases.

There may be a generalized loss of rim with generalized enlargement of the cup in a sequential manner or there may be a focal loss. The focal damage is when there is notching or focal rim thinning.

The notch is defined as localized defect in the NRR on the cup side of the rim. The rim:disk ratio in the area of the notch is smaller than the rim:disk ratio immediately adjacent to the notch and the circumferential extent of the notch occupies less than or equal to 60°, i.e. 2 contiguous clock hours (Fig. 8).

**Figs 9A to c:**
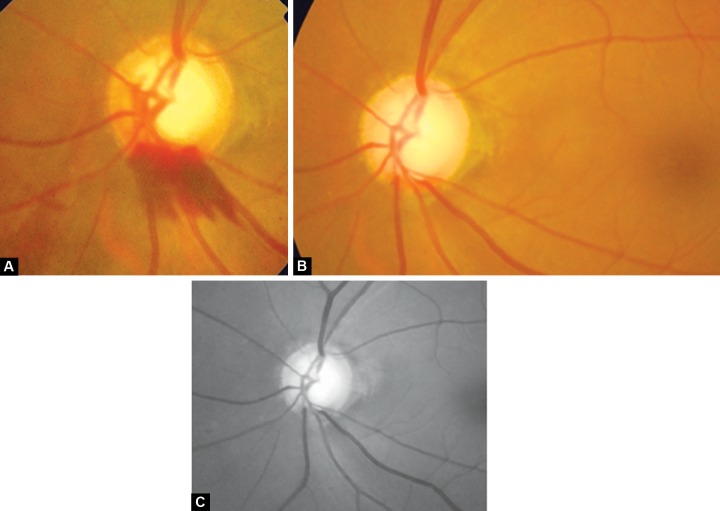
(A) Disk hemorrhage, (B) after 3 months–hemorrhage replaced by wedge shaped RNFL defect inferiorly, (C) red free picture of same highlighting RNFL

### Disk Hemorrhage

A disk hemorrhage is a splinter or fame-shaped hemorrhage, with feathered edges, oriented radially and perpendicular to the disk margin. These are located in the prelaminar area of the optic disk and in the adjacent superficial retinal nerve fiber. Some are round and blotchy because they are situated in deeper parts of the disk and, occasionally, when the cup is large, a hemorrhage can be seen on the lamina cribrosa itself.

**Fig. 10:**
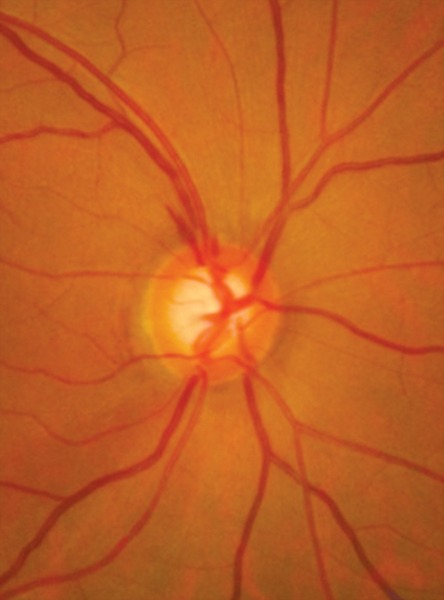
Splinter hemorrhage typical of glaucoma

**Fig. 11:**
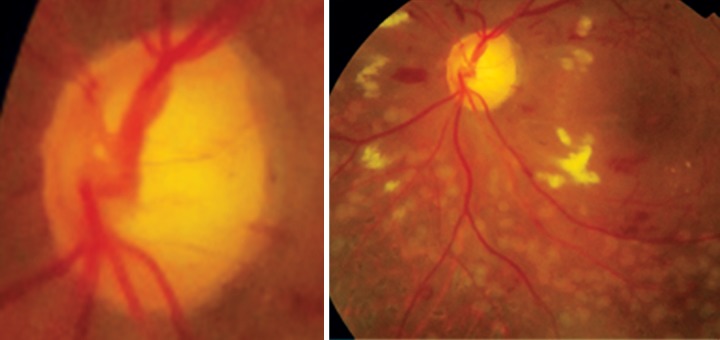
Hemorrhage at disk due to diabetic retinopathy

It is located within one disk diameter from the optic disk border and one should rule out presence of optic disk edema, papillitis, diabetic retinopathy, central or branch retinal vein occlusion, or any other retinal disease associated with hemorrhage (Figs 9A, 10 and 11).

The prevalence of disk hemorrhage ranges from 0.6 to 1.4% in the normal population and from 1.9 to 16.9% in subjects with glaucoma.^16^ The cumulative incidence of optic disk hemorrhages is reported as 0.5% per year in ocular hypertensives and 2.5% per year in eyes after the development of primary open angle glaucoma (POAG).^17^ Reported incidence in primary angle closure glaucoma (PACG) is 5.4% in 9-year follow-up.^18^ The reported incidence in APEDS is 9.8% in POAG group.^19^

Optic disk hemorrhages are more common in the eyes with normal pressure glaucoma (5-9%) than in eyes with POAG (4. 3%) which are more frequent than in eyes with secondary open angle glaucoma (1.2%). The frequency of bilateral hemorrhages reportedly ranges from 5.6 to16.7%, and that of recurrent hemorrhages from 45.6 to 53.6%.

The disk hemorrhages are transient and the average duration is 12.8 ± 8.1 weeks (range: 1-42) (Figs 9B and C).

To detect disk hemorrhage it is necessary to look for them and document presence or absence of them. It is noted that optic disk photographs detect hemorrhage more often than clinical examinations.^17^ A dilated fundus examination increases the chances of detecting them.

In POAG disk hemorrhages tend to concentrate at the temporal upper and lower poles, which are sites of early glaucomatous rim loss.^16^ Large vertical C:D ratio and pseudoexfoliation may be independent risk factors for disk hemorrhage. Other associations are migraine, diabetes, aspirin use and systemic hypertension.^16^

### Pathogenesis

Ischemic microinfarction at the optic disk,^20^ structural changes at the level of lamina cribrosa^21^ are suggested as the pathogenesis of disk hemorrhage.

The higher frequency noted in normal tension glaucoma is attributed to the higher transmural pressure. Transmural pressure difference is inversely correlated with the intraocular pressure. If a small blood vessel ruptures, the amount of blood leaking out of the vessel into the surrounding tissue will be larger because of the higher pressure gradient. This suggests that larger leaks are expected in normal tension glaucoma and since they take longer to get absorbed hence chances of picking them up are also higher.^22^

### Significance

Disk hemorrhage is a poor prognostic sign in glaucoma, often preceding nerve fiber layer damages, optic disk changes or visual field defects that are associated with progressive glaucomatous damage.

The ocular hypertension treatment study (OHTS)^13^ shows that optic disk hemorrhages are a predictive factor for the development of POAG in patients with OHT. In the Collaborative Normal Tension Glaucoma Study (CNTGS) and the Early Manifest Glaucoma Trial (EMGT), glaucomatous eyes with disk hemorrhages experienced significantly more visual field progression during follow-up than eyes without hemorrhages.

### Retinal Nerve Fiber Layer

In normal eyes the RNFL is most visible in the temporal inferior and temporal superior sectors and least visible in the nasal sectors.^2^ This has correlation with the histology of the RNFL which is thicker in inferior and superior peripapillary areas than the temporal and nasal. The NRR is wider and the lamina cribrosa pores and diameter of the retinal arterioles is larger in these areas. The visibility of RNFL decreases with age.

**Fig. 12:**
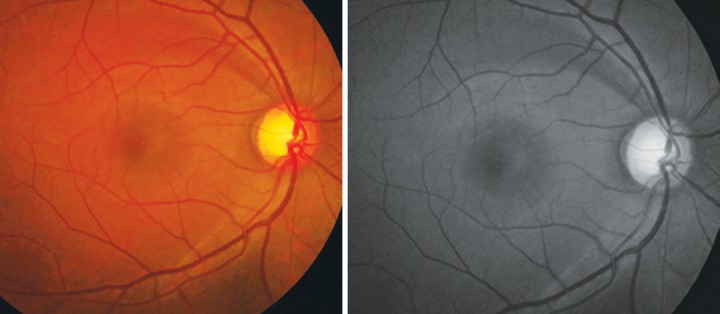
RNFL defect superior. Better seen in red free photograph below

**Fig. 13:**
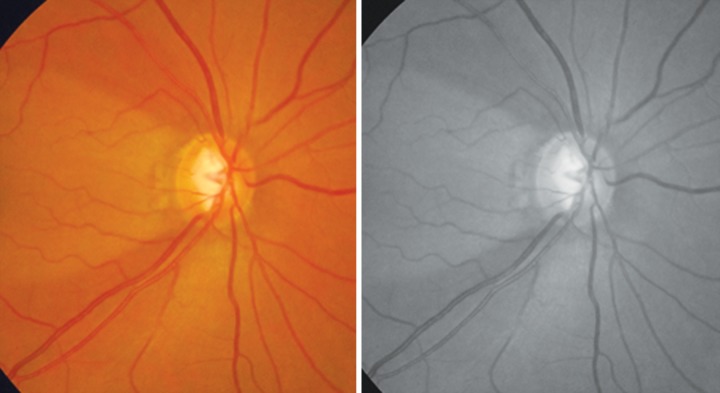
RNFL defect superior and inferior. Also note presence of bipolar notch. Red free photo below

To examine the RNFL dilate the pupil and see the fundus with a noncontact lens in red free illumination of the slit lamp. The normal pattern of the fiber bundles can be detected as bright striations in the retinal refex. If in a fundus the RNFL is markedly better detectable in the temporal superior sector one can carefully observe the temporal inferior region to find a loss of RNFL. Also observe the vessels as the retinal vessels are normally embedded in the retinal nerve fibers and when there is diffuse RNFL loss the vessels are covered only by the thin inner limiting membrane so are better visible.

The RNFL defects could be localized or generalized depending on the stage of the disease and because localized defects are very rare in normal eyes they are highly specific for optic nerve damage. But localized defects are not pathognomic of glaucoma and can occur in other types of optic disk atrophies. In glaucoma, they are more common in normal tension glaucoma followed by POAG and then secondary open angle cases.

A localized defect is defined as a wedge-shaped defect running toward or touching the optic disk border occupying not more than 60° of the circumference of the disk. This has to be differentiated from a spindle like narrow dark area or a pseudodefect.

The RNFL defects are usually first seen in the temporal inferior and superior sectors (Figs 12 and 13).

The number of localized defects is more in early and moderate glaucoma and rare in advanced optic nerve damage.^23^

Quigley et al^24^ described these RNFL changes to be sensitive indicators of early optic nerve damage seen earlier than optic disk changes.

RNFL defects are seen in areas where the NRR is thin, notched or where the disk hemorrhage was located. The localized defect is seen after 6 to 8 weeks of the appearance of disk hemorrhage^25^ (Fig. 9).

Examination of the RNFL can detect early glaucomatous changes even before perimetry and is of importance in detection in pseudonormal but glaucomatous minicup in minidisks^26^ and to classify an eye with pseudoglaucomatous but normal large cup in a large disk. Differentiation of glaucomatous from nonglaucomatous also can be enhanced by examination of the RNFL.

### Peripapillary Atrophy

The more peripheral zone is called alpha zone and is characterized by irregular hypopigmentation and hyperpigmentation and by thinning of the overlying chorioretinal layer. Zone beta is located closer to the optic disk border and is more distinctive because of the visible sclera and visible large choroidal vessels. The alterations in peripapillary region can be acquired and can progress in conjunction with progressive glaucomatous optic atrophy. But it is less certain that distinguishing the peripapillary atrophy either enhances the recognition of glaucoma or presages its progression.

Some maintain that progressive changes in the beta zone are IOP independent, others have associated pronounced alterations with normal tension glaucoma and age-related paging contrast to glaucomatous eyes, eyes with nonglaucomatous optic nerve atrophy; including eyes after arteritic anterior ischemic optic neuropathy, do not show an enlarged peripapillary atrophy.

### Certain Disk Anomalies which can be Confused with Glaucoma

Careful clinical examination of the optic nerve head using all the above-mentioned parameters can help differentiate normal disk from glaucomatous (Table 1). All these should be evaluated in each patient and documented to evaluate change over time.

**Table 1:** **Table 1:** Differentiation between morning glory disk anomaly and disk coloboma^27^

Morning glory (Fig. 14)		Optic disk coloboma (Fig. 15)	
Optic disk lies within the excavation		Excavation lies within the optic disk	
Symmetrical defect (disk lies centrally)		Asymmetrical (excavation usually inferior)	
Central glial tuft		No central glial tuft	
Severe peripapillary pigmentary disturbance		Minimal peripapillary pigmentary disturbance	
Anomalous retinal vasculature		Normal retinal vasculature	
More common in females, rare in blacks		No sex or racial predilection	
Rarely familial		Often familial	
Rarely bilateral		Often bilateral	
No iris, ciliary or retinal colobomas		Iris, ciliary and retinal colobomas common	
Rarely associated with multisystem genetic disorder		Commonly associated	
Basal encephalocele common		Basal encephalocele rare	

**Fig. 14:**
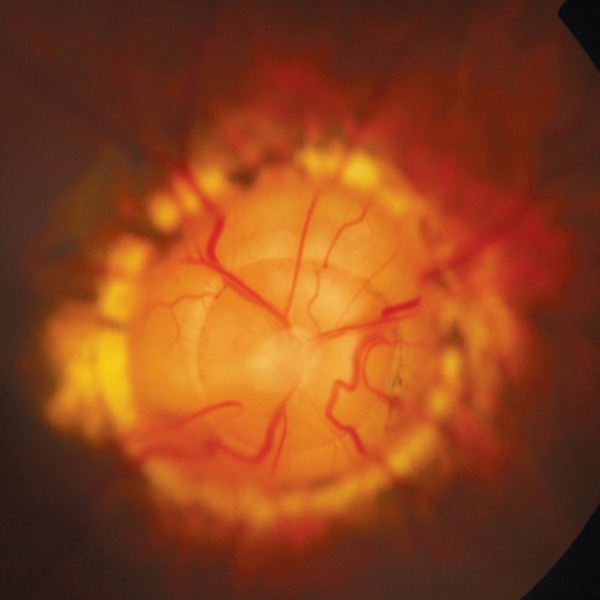
Morning glory syndrome

**Fig.15:**
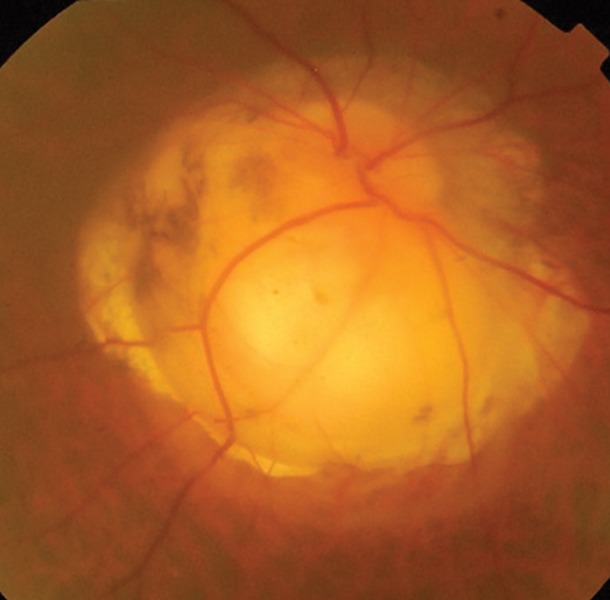
Optic disk coloboma
